# Thymoquinone Promotes Pancreatic Cancer Cell Death and Reduction of Tumor Size through Combined Inhibition of Histone Deacetylation and Induction of Histone Acetylation

**DOI:** 10.1155/2016/1407840

**Published:** 2016-12-26

**Authors:** Daniel Relles, Galina I. Chipitsyna, Qiaoke Gong, Charles J. Yeo, Hwyda A. Arafat

**Affiliations:** ^1^Departments of Surgery, Jefferson Pancreatic, Biliary and Related Cancer Center, Thomas Jefferson University, Philadelphia, PA, USA; ^2^Department of Biomedical Sciences, University of New England, Biddeford, ME, USA

## Abstract

Pancreatic ductal adenocarcinoma (PDAC) is virtually therapy-resistant. As noninvasive lesions progress to malignancy, the precursor period provides a window for cancer therapies that can interfere with neoplastic progression. Thymoquinone (Tq), a major bioactive component of essential oil from* Nigella sativa*'s seeds, has demonstrated antineoplastic activities in multiple cancers. In this study, we investigated antineoplastic potential of Tq in human PDAC cell lines, AsPC-1 and MiaPaCa-2. Tq (10–50 *μ*M) inhibited cell viability and proliferation and caused partial G2 cycle arrest in dose-dependent manner in both cell lines. Cells accumulated in subG0/G1 phase, indicating apoptosis. This was associated with upregulation of p53 and downregulation of Bcl-2. Independently of p53, Tq increased p21 mRNA expression 12-fold. Tq also induced H4 acetylation (lysine 12) and downregulated HDACs activity, reducing expression of HDACs 1, 2, and 3 by 40–60%. In vivo, Tq significantly reduced tumor size in 67% of established tumor xenografts (*P* < 0.05), along with increased H4 acetylation and reduced HDACs expression. Our results showed that Tq mediated posttranslational modification of histone acetylation, inhibited HDACs expression, and induced proapoptotic signaling pathways. These molecular targets demonstrate rationale for using Tq as a promising antineoplastic agent to prevent postoperative cancer recurrence and to prolong survival of PDAC patients after surgical resection.

## 1. Introduction

*Nigella sativa* is a herb used in traditional medicine in many Middle Eastern countries to treat a broad array of diseases, including asthma, diabetes, hypertension, headache, eczema, and various gastrointestinal ailments. Thymoquinone (Tq), the most abundant constituent of* Nigella sativa* seed's essential oil extract, has been demonstrated to have antioxidant and anti-inflammatory activity through mechanisms that are still being defined [1–3]. Additionally, Tq has been reported to display antineoplastic activity in multiple cancers, including prostate, colorectal, breast, sarcoma, and leukemia [1–3]. Tq has been shown to inhibit cancer cells proliferation, arrest cancer cell cycle progression, induce proapoptotic effects, and enhance cytotoxic activity of chemotherapeutic agents [3–6]. Implicated molecular targets include Bcl-2, caspases, PPARs, NF-kB, STAT3, MAPK, Akt, and ROS [3]. Proapoptotic activity of Tq is revealed through p53-dependent and independent pathways. In murine model xenografts Tq has been shown to suppress tumor growth, likely through angiogenesis inhibition [4]. Tq was shown to inhibit cell migration and invasion in breast cancer cells in a dose-dependent manner [3]. Finally, Tq has been shown to mitigate some of the drug-related toxicities associated with several chemotherapeutic agents [3, 5, 6]. Small study of safety and clinical activity of Tq in humans found no adverse effects with doses up to 2600 mg/day [3].

In eukaryotic cells, DNA is tightly wound to form nucleosomes around core histone proteins. Regulation of gene expression is controlled through acetylation and deacetylation of histones, which are modified in posttranslational manner through histone acetyltransferases (HATs) and histone deacetylases (HDACs). Inhibition of HDACs leads to histones acetylation and subsequent changes in gene expression, and it has been shown to inhibit cell cycle progression and promote apoptosis in cancer cells [7]. HDAC inhibitors have been reported to induce p21 expression in p53-independent manner [8]. We recently have shown that Tq can act as a novel inhibitor of proinflammatory pathways in PDAC cells, and this is mediated through significant downregulation of constitutive and TNF-*α*-activated NF-*κ*B [9]. Similar results were obtained using Trichostatin A (TSA), a well-established HDAC inhibitor, thus showing that Tq can act as HDAC inhibitor [9]. However, it is yet to be determined whether Tq's antineoplastic activity involves histone acetylation and deacetylation.

In this study, we explored the antineoplastic potential of Tq in PDAC cells. In addition to studies investigating the relationship between Tq and p21 expression, we evaluated Tq's effect on histone acetylation/deacetylation in vitro and in vivo.

## 2. Materials and Methods

### 2.1. Cell Culture

PDAC cell lines, AsPc-1, MiaPaCa-2, and Hs766T, were purchased from ATCC. Cells were cultured in DMEM supplemented with 10% fetal bovine serum in humid atmosphere of 5% CO_2_. Cells were treated with Tq (10–50 *μ*M) (Sigma-Aldrich). Tq concentrations were selected based on dose-response studies.

### 2.2. MTT Assay

Cells were grown in 48-well plates and incubated in full growth media at 37°C and 5% CO_2_. After treatment with Tq, cell viability was examined using MTT (Methylthiazolyldiphenyl-tetrazolium bromide) conversion assay as previously described. Briefly, MTT (Sigma-Aldrich) was added to the wells (500 *μ*g/mL). After 4 hours formazan crystals were solubilized in DMSO, and the optical density was measured at 570 nm. Optical density directly correlated with viable cells quantity. Experiments were made in triplicate and repeated 3 times.

### 2.3. Flow Cytometric Analysis of DNA Content

Cells, seeded in 100 mm dishes at a density of 7.5 × 10^5^ cells/dish, were treated with Tq and incubated for 24 or 48 h. Cells were harvested, washed with phosphate-buffered saline (DPBS), permeabilized with 70% ethanol, treated with 1% RNase, and stained with propidium iodide (PI) at 100 *μ*g/mL. Flow cytometric analyses were carried out in a fluorescence-activated sorter (FACStar plus; Becton Dickinson) with a 360 nm Argon-Iron laser. 10,000 events per sample were collected and analyzed using the Cell Fit analysis program (Becton Dickinson).

### 2.4. Annexin V-FITC Staining

After treatment with Tq, cells were washed with DPBS buffer and removed from the growth plates. Cells were collected by centrifugation at 500*g* for 5 min, and Annexin V-FITC reagent (BD Bioscience) with or without PI counterstain was used to resuspend the pellet. After incubation in darkness for 15 min at room temperature cells were analyzed by flow cytometry. FITC (FL1, BP525/5 nm) and PI (FL3, BP 610/5 nm) were collected in logarithmic mode. Analysis was performed using Beckman Coulter Elite software.

### 2.5. RNA Extraction and qRT-PCR

Total RNA was isolated using Tri-Reagent (Life Technologies). RNAs were quantified and cDNAs were synthesized using ImProm-II™ Reverse Transcription System (Promega). TaqMan gene expression assays were purchased from Applied Biosystems. GAPDH was used as a housekeeping gene. cDNAs were subjected to real time qPCR using HotStart-IT or VeriQuest Probe qPCR Master Mixes (Affymetrix) and TaqMan technology (7500 Sequence Detector, Applied Biosystems). The relative mRNA levels were quantified using Applied Biosystems software.

### 2.6. Protein Isolation and Western Blot Analysis

Cell or tissue lysates were analyzed as described elsewhere [10]. Cells were lysed in modified RIPA lysis buffer, and protein concentrations in the supernatant were determined using BCA protein assay reagent (Pierce). Equal protein quantities (25 *μ*g) were run in 10% SDS-polyacrylamide slab gels, transferred to polyvinylidene difluoride membranes, and incubated with primary antibodies, acetylated H4 (Active motif), and anti-*β* actin (Chemicon), both diluted in PBST. After incubation with secondary antibodies, protein bands were visualized with enhanced chemiluminescence reagents (ECL Plus Western Blotting Detection System, Amersham Pharmacia Biotech).

### 2.7. Histone Deacetylases (HDACs) Activity

To determine HDACs activity in PDAC cells after Tq treatment we used a colorimetric HDAC activity assay kit (Biovision). HDAC colorimetric substrate (acetylated lysine side chain) was incubated with nuclear extract of PDAC cells after treatment with Tq. Deacetylation of the substrate sensitized the substrate, which upon treatment with the Lysine Developer produced a chromophore. The chromophore absorbance at 405 nm that directly correlated with HDAC activity was determined using Synergy HT multidetection microplate reader (BioTeck).

### 2.8. Tq Toxicity in Mice

Mice (8-week-old) were divided into groups of 10 each and Tq toxicity was determined after daily intraperitoneal injections for 20 consecutive days. The doses of Tq were 5, 10, 20, or 30 mg/kg body weight. Tq dilutions in isotonic saline (0.9%) were prepared and control animals received the same percentage of ethanol diluted in saline.

### 2.9. Xenograft Model

Animal studies were performed after approval of the protocol by IACUC at Thomas Jefferson University Hospital. Xenograft model for PDAC was generated in 4-week-old male nude mice (Crl : Nu/Nu-muBR) weighing 20–22 g (Charles River Laboratories). Suspensions of AsPC-1 or Hs766T PDAC cells (5 × 10^6^ in 0.2 mL PBS) were injected subcutaneously into the flank of 4–6-week-old male mice. Animals were kept in light- and temperature-controlled environment and provided with food and water ad libitum. Tumor size was determined daily. Intraperitoneal treatment with Tq (5–30 mg/kg) or 0.1% methanol in physiologic saline three times per week was started when subcutaneous tumors reached a diameter of 1 cm. After 5 weeks animals were sacrificed, tumor sizes were measured, and tumors were processed for evaluation of HDAC expression levels by real time qPCR.

### 2.10. Statistical Analysis

All experiments were performed 4 to 6 times. Data were analyzed for statistical significance by ANOVA with post hoc Student's *t*-test analysis. These analyses were performed with assistance of a computer program (JMP 5 Software SAS Campus Drive). Differences were considered significant at *P* ≤ 0.05. Tumor sizes were estimated for significant differences by the Manny–Whitney rank sum test.

## 3. Results

### 3.1. Tq Induces a Dose-Dependent Reduction of PDAC Cell Growth

**Figure 1:**
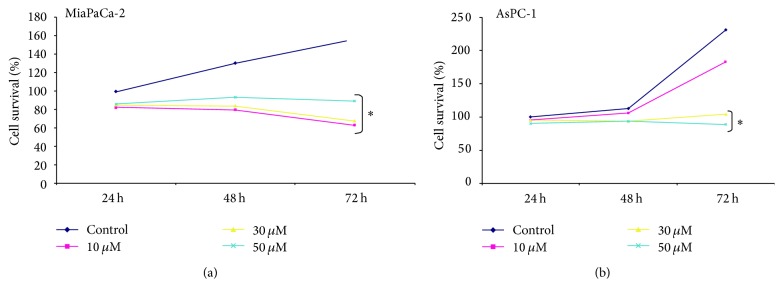
Effect of Tq on PDAC cell proliferation. (a) MiaPaCa-2 and (b) AsPc-1 cells were exposed to Tq (10–50 *μ*M) during 24, 48, and 72 h. ^*∗*^*P* < 0.05 versus control, using one-way repeated ANOVA with subsequent all pairwise comparison procedure by Student's *t*-test.

Cell viability was significantly reduced by Tq as a function of both concentration and time, in both cell lines, MiaPaCa-2 (Figure 1(a)), and AsPC-1 (Figure 1(b)). For example, cell survival for MiaPaCa-2 cells treated with 50 *μ*M Tq for 24, 48, and 72 h was 82%, 80%, and 62%, respectively. Differences in cell survival at increasing concentrations became more pronounced with longer treatment times. These results confirm that Tq inhibits PDAC cell proliferation.

### 3.2. Tq Induces Accumulation of PDAC Cells in the PreG1 Peak

**Figure 2:**
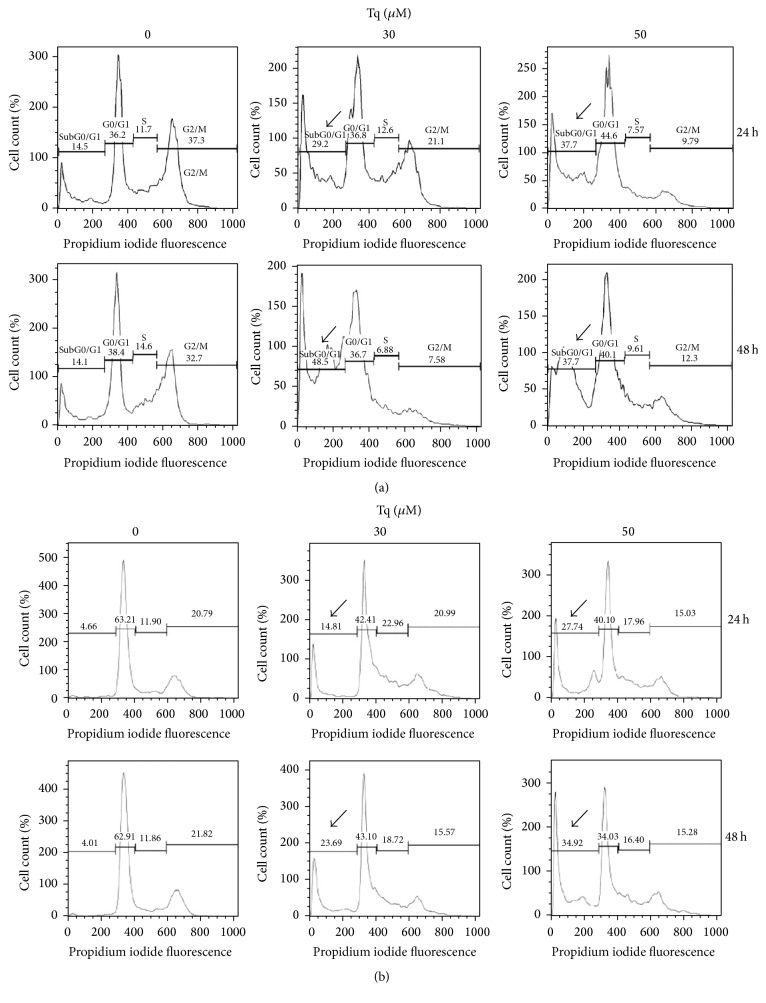
Cell cycle profiles by flow cytometry: (a) MiaPaCa-2 and (b) AsPc-1 cells. Tq induced accumulation of the cells in preG1 phase. The data shown are typical of one of three independent experiments. DNA histograms show evident accumulation of hypodiploid cells at preG1 peak within 24 h indicating accumulation of dead and apoptotic cells. At 30 *μ*M of Tq the percentage of apoptotic cells increased to 29% at 24 h and reached 49% at 48 h (a), while it increased to 15% in AsPC-1 cells after 24 h and reached 24% after 48 h (b). Data were quantified from three experiments, ^*∗*^*P* < 0.05, using one-way repeated ANOVA with subsequent all pairwise comparison procedure by Student's *t*-test.

Cell cycle profiles were monitored by flow cytometric analysis of PI stained cellular DNA content at 24 and 48 h after treating MiaPaCa-2 and AsPC-1 cells with Tq (30 and 50 *μ*M). DNA histograms show evident accumulation of hypodiploid cells at preG1 peak within 24 h indicating accumulation of dead and apoptotic cells. At 30 *μ*M of Tq the percentage of apoptotic cells increased to 29% at 24 h and reached 49% at 48 h (Figure 2(a)), while it increased to 15% in AsPC-1 cells after 24 h and reached 24% in after 48 h (Figure 2(b)).

### 3.3. Tq Induces Late Apoptosis

**Figure 3:**
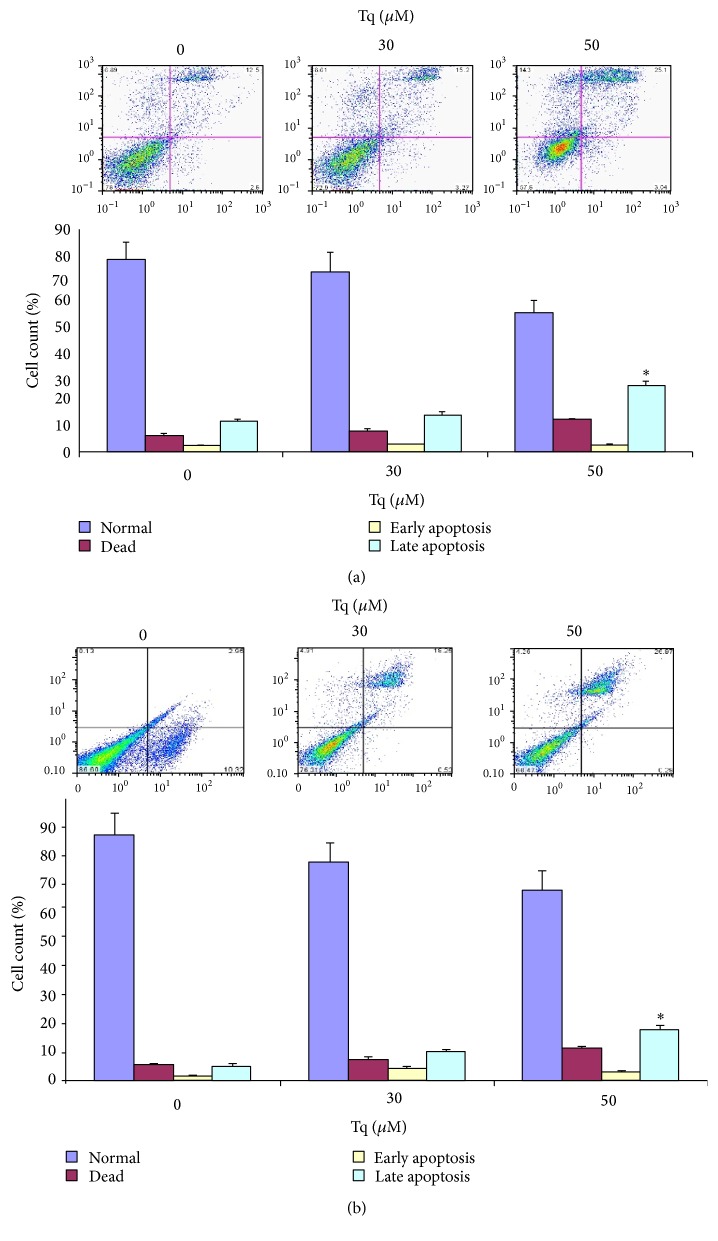
Apoptosis in Tq-induced PreG1 fraction. (a) MiaPaCa-2 and (b) AsPc-1 cells were treated with Tq (30 and 50 *μ*M) for 24 h, stained with Annexin V and PI, and apoptosis was analyzed by flow cytometry. The upper right quadrants represent apoptotic cells, Annexin V positive and PI negative. Quantification of cell numbers in histogram shows significant increase in the number of late apoptotic cells. Data were quantified from four experiments ^*∗*^*P* < 0.05, using one-way repeated ANOVA with subsequent all pairwise comparison procedure by Student's *t*-test.

Analysis of the number of cells in the histogram revealed that the number of late apoptotic cells was significantly increased with concomitant reduction in the number of live cells in both MiaPaCa-2 (Figure 3(a)) and AsPC-1 cells (Figure 3(b)). These findings confirmed that Tq-induced PDAC cell number increases in preG1 fraction were due to apoptosis.

### 3.4. Tq-Induced Apoptosis Is Accompanied by Regulation of Apoptosis-Related Genes

**Figure 4:**
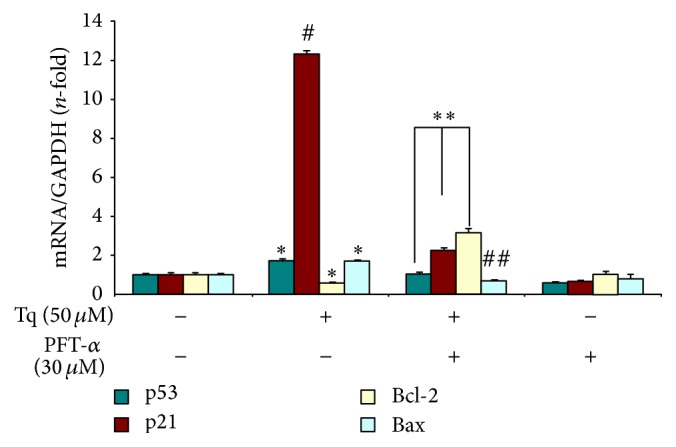
Real time qPCR analysis shows that Tq (50 *μ*M) significantly increased p53, p21, and Bax mRNA expression and downregulated Bcl-2 mRNA expression in MiaPaCa-2 cells. Cells were also pretreated with p53 inhibitor, PFT-*α*, for 1 h prior to addition of Tq. Values are expressed as mean ± SEM of three experiments. ^*∗*^*P* < 0.05  ^#^*P* < 0.02 versus control levels ^*∗∗*^*P* < 0.05  ^##^*P* < 0.02 versus Tq treated values, using one-way repeated ANOVA with subsequent all pairwise comparison procedure by Student's *t*-test.

To analyze the molecular mechanisms of Tq-mediated apoptosis, we explored the expression of pro- and antiapoptotic genes in MiaPaCa-2 cells. Real time qPCR analysis indicated that Tq induced a dose-dependent increase in Bax mRNA expression (Figure 4). Tq dose-dependently downregulated Bcl-2 mRNA expression and, as a result, increased Bax/Bcl-2 mRNA ratio. In addition, Tq induced 2-fold increase in p53 mRNA expression after 24 h of treatment and 12-fold increase in p21 mRNA expression after as early as 3 h of treatment (Figure 4). Cells were also pretreated with p53 inhibitor, PFT-*α*, for 1 h prior to addition of Tq. This treatment abolished p53 induction, decreased Bax/Bcl-2 mRNA ratio, and reduced expression of p21 mRNA from 12-fold to 2-3-fold. These data provided the evidence of p53-independent upregulation of p21 by Tq. Since many HDAC inhibitors have been shown to stimulate p21 signaling regardless of the mutation status of p53, we investigated whether Tq can act as HDAC inhibitor.

### 3.5. Tq Significantly Reduces the Expression of HDACs 1, 2, and 3 mRNA Expression and HDAC Activity

**Figure 5:**
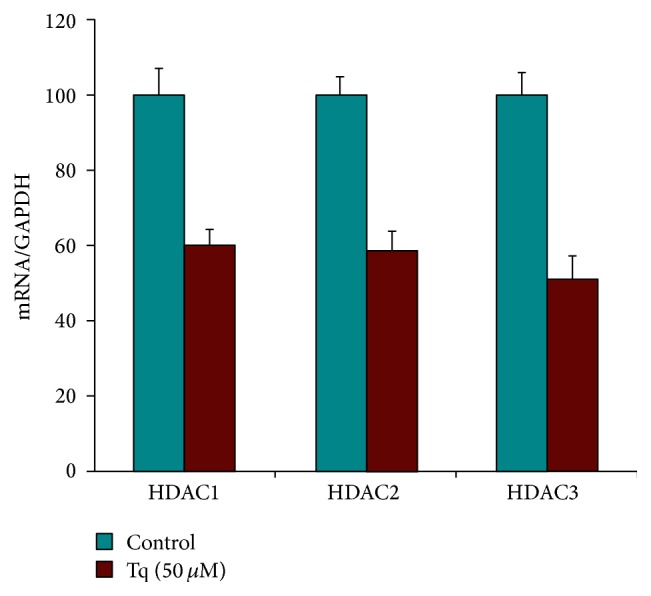
Real time qPCR analysis of MiaPaCa-2 cells showing significant reduction of HDACs 1, 2, and 3 levels upon treatment with Tq (50 *μ*M). ^*∗*^*P* < 0.05 versus control levels, using one-way repeated ANOVA with subsequent all pairwise comparison procedure by Student's *t*-test.

**Figure 6:**
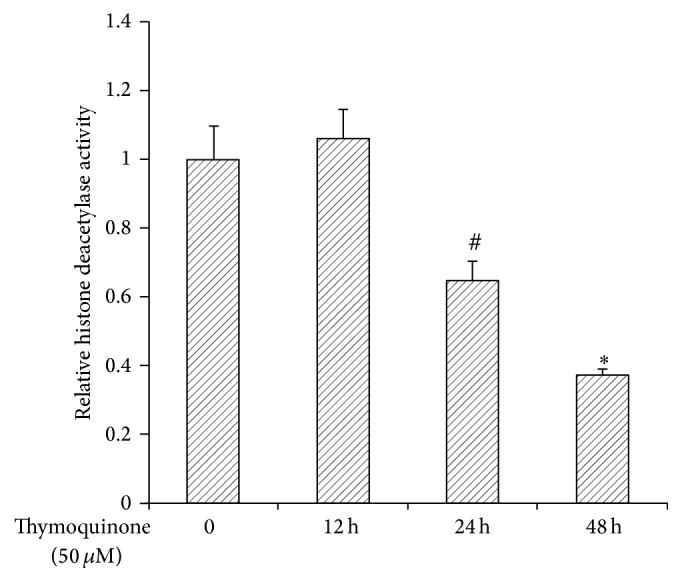
MiaPaCa-2 cells were treated with or without Tq (50 *μ*M) for 12–48 h. HDACs activity decreased significantly at 24 and 48 h. Values are expressed as mean ± standard deviation of three different observations. ^#^*P* < 0.05  ^*∗*^*P* < 0.002 versus control levels, using one-way repeated ANOVA with subsequent all pairwise comparison procedure by Student's *t*-test.

To determine whether Tq acts through inhibition of HDACs, we evaluated the expression of HDACs 1–6 by real time qPCR. As seen in Figure 5, Tq (50 *μ*M) reduced the expression of HDACs 1–3 by 40–50%. Tq (50 *μ*M) time-dependently reduced HDACs activity by 60% (Figure 6).

### 3.6. Tq Dose-Dependently Increases Histone Acetylation

**Figure 7:**
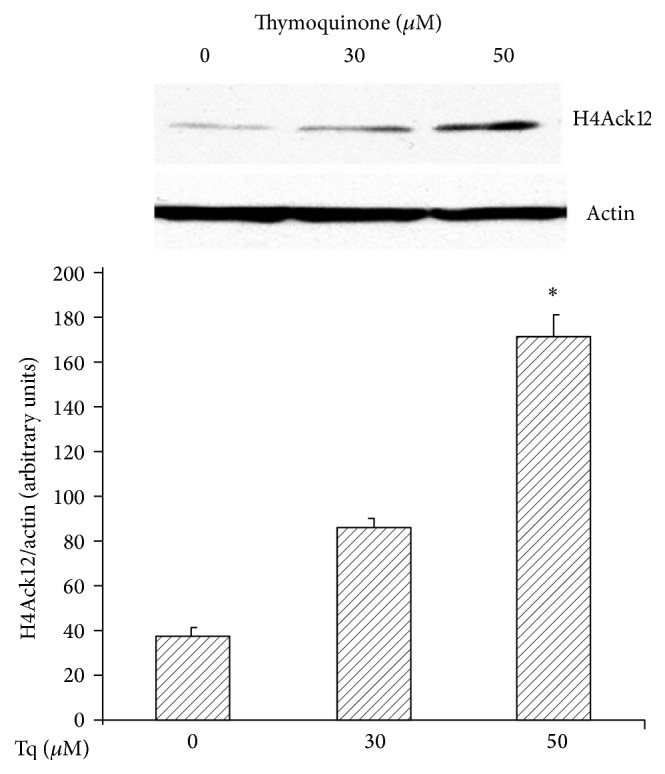
Representative Western immunoblot of nuclear extracts from MiaPaCa-2 cells. H4 Ac K12 protein is expressed as 8 kDa band. Significant increase of H4 Ac K12 protein is seen in cells treated with Tq (30–50 *μ*M) after 48 h. Data are means ± SEM. ^*∗*^*P* < 0.05 versus control untreated cells.

To assess whether histone acetylation is involved in Tq mode of action, we evaluated the acetylation of lysine 12 in histone 4 (H4 Ac-K12) by Western blotting. Nuclear extracts from Tq treated (30–50 *μ*M) MiaPaCa-2 cells showed dose-dependent increased acetylation after 24 h (Figure 7). These data indicate that Tq not only reduces HDAC expression and activity but also increases histone acetylation.

### 3.7. Tq Significantly Reduces Tumor Size in Human PDAC Xenografts

**Figure 8:**
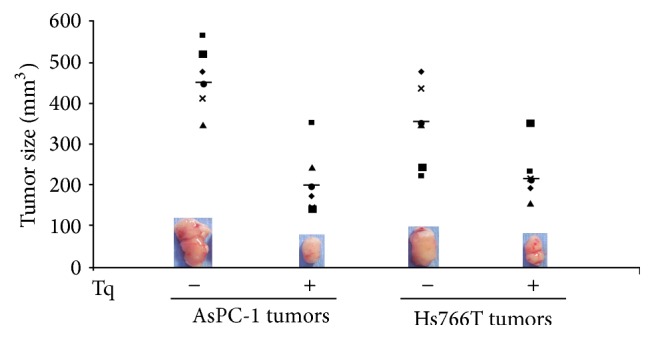
Xenografts generated in 4-week-old male nude mice using AsPC-1 and Hs766T cells. When tumors reached 1 cm, animals were treated with Tq (30 mg/kg body weight i.p. for 5 weeks). Tumor size was significantly (*P* < 0.05) shrunken in 67% of the animals.

**Figure 9:**
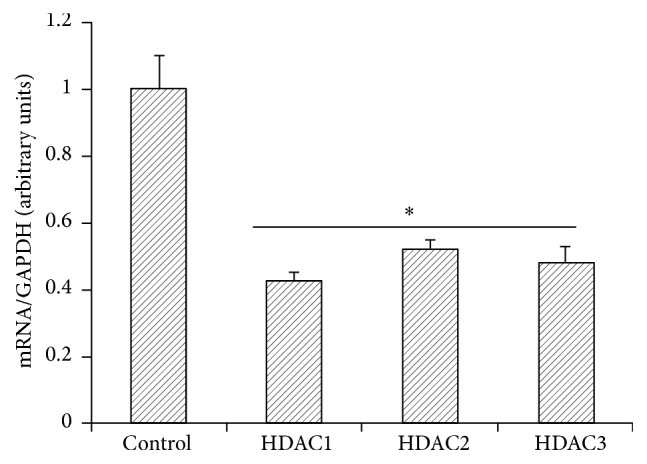
Real time qPCR of Tq treated xenografts showing significant reduction of HDACs 1, 2, and 3 mRNA expression in AsPC-1 cells. Data are means ± SEM. ^*∗*^*P* < 0.05 versus control untreated tumors.

Next, we tested the effect of different doses of Tq in human PDAC xenografts. Xenografts were generated in 4-week-old male nude mice (Crl : Nu/Nu-muBR) weighing 20–22 g (Charles River laboratories). Human PDAC cell lines (AsPC-1 and Hs766T) were utilized. When the tumors reached 1 cm, animals were treated with different doses of Tq (5–30 mg/kg body weight). We show here data from animals that were treated with Tq at 30 mg/kg body weight for 5 weeks. Tumor size was significantly (*P* < 0.05) shrunken in 67% of the animals (Figure 8). Analysis of HDACs in the tumors indicated that Tq had significantly reduced the expression of HDACs 1, 2, and 3 mRNA when compared to vehicle-treated controls (Figure 9).

## 4. Discussion

Pancreatic ductal adenocarcinoma (PDAC) is still an urgent clinical problem because of its aggressive nature and resistance to chemotherapy and radiation. In this study, we show that thymoquinone (Tq), the active ingredient of the Middle Eastern herb,* Nigella sativa*, significantly inhibits PDAC cell growth and triggers induction of different proapoptotic signaling pathways in human PDAC cells in vitro and in vivo. This study is also the first to show that Tq mediates posttranslational modification of histone acetylation and inhibition of HDACs expression.

Tumor suppressor gene p53 is a sensor of cellular stress and is a critical activator of intrinsic pathway of apoptosis. Stimulation of p53 can initiate apoptosis by activating proapoptotic Bcl-2 family member, such as Bax, and repressing antiapoptotic Bcl-2 proteins, such as Bcl-2 and Bcl-xl. Bax undergoes conformational changes and subsequently translocates to mitochondria, where it inserts into the outer membrane as oligomers, resulting in the release of cytochrome *c* and apoptosis [11]. Bcl-2/Bcl-xl form heterodimers with Bax and prevent its insertion into the mitochondrial membrane. Therefore, the ratio of Bcl-2/Bcl-xl to Bax is critical for the determination of the apoptotic threshold. Studies have shown that deregulation of Bax/Bcl-2/Bcl-xl contributes to the development, growth, and expansion of PDAC [12], and increased Bax and reduced Bcl-2/Bcl-xl significantly increase the sensitivity to chemotherapeutic drugs [13, 14]. Bcl-xl is believed to be an ideal target for PDAC therapy as it is constitutively overexpressed in PDAC cell lines that are highly resistant to Fas- and TRAIL-mediated apoptosis [15]. In our studies, we showed that Tq (100 *μ*M) induced a significant increase in Bax/Bcl-2 mRNA expression ratio and caused apoptosis in PDAC cells.

Cells also respond to activation of p53 tumor suppressor by undergoing cell cycle arrest through activation of p21 expression. Our results demonstrated that there was a significant increase (12-fold) in p21 expression, without an equivalent increase in p53 expression (2-fold) in PDAC cells treated with Tq for as early as 3 h. This suggests that Tq induces p21 expression, partially in p53-independent manner. Since many HDAC inhibitors have been shown to stimulate p21 signaling regardless of the mutation status of p53 [8, 16], we investigated whether Tq could act as HDAC inhibitor.

Histone acetylation and deacetylation (epigenetic regulation) is the mechanism of regulating gene expression. In tumor cells, acetylation/deacetylation balance becomes disrupted. As histones are modified chromatin structure and subsequent gene expression change. Histone acetyltransferases (HATs) decondensate chromatin and loosen the folded nucleosome, which enables the binding of transcription factors to promoter sites [8, 17, 18]. This effectively activates genes expression. Histone deacetylases (HDACs) have the opposite mechanism and effect, resulting in genes silencing. Hypoacetylation, due to either decreased HAT or increased HDAC activity, results in silencing of tumor suppressor genes. Histone hypoacetylation or HDACs overexpression has been linked to the development of prostate, breast, ovarian, colon, and gastric cancer [19]. It has been shown that HDACs are overexpressed in PDAC [20] and that this, together with HIF-1a overexpression, is associated with worsening of survival prognosis [21].

In contrast to genetic mutations, which are almost impossible to reverse, epigenetic changes are potentially reversible. This implies that they are amenable to pharmacological interventions. HDAC inhibitors (HDAC-i) have shown to display antineoplastic activity in multiple tumor types, inhibiting cell growth and inducing apoptosis [17, 19]. In 2006, first HDAC-i, vorinostat (Zolinza), was approved as single-agent use in T cell lymphoma [18]. Today, several epigenetic drugs are already approved by the FDA and the EMEA for cancer treatment and around ten HDAC-is are in clinical development [22]. In PDAC cell lines usage of HDAC-is in combination with conventional chemotherapies has demonstrated improved antitumor activity as compared to conventional chemotherapy alone [23–25]. In vivo, however, this has not been shown. Phase II study of gemcitabine in combination with tacedinaline (a known HDAC-i) showed no improvement in patient response rate or survival compared to gemcitabine alone [26]. Thus search for effective HDAC-is continues.

**Figure 10:**
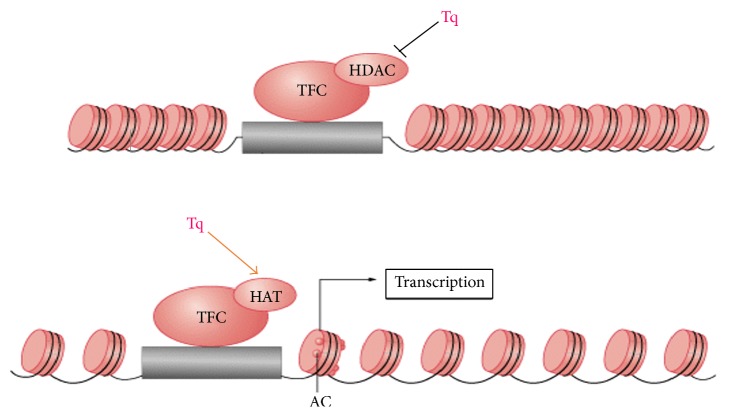
Tq actions as HDAC inhibitor. Combined inhibition of histone deacetylation and induction of histone acetylation.

Our study demonstrated that Tq could act as HDAC-i and could potently induce apoptosis in PDAC cells and affect the epigenetic state of histone through induction of histone acetylation and inhibition of histone deacetylation (Figure 10). Our data suggest that Tq, as a multitarget agent, has a clinical potential that combines anti-inflammatory and proapoptotic epigenetic modification modes of action.
